# Pyoderma outbreak among kindergarten families: Association with a Panton-Valentine leukocidin (PVL)-producing *S*. *aureus* strain

**DOI:** 10.1371/journal.pone.0189961

**Published:** 2017-12-19

**Authors:** Rasmus Leistner, Axel Kola, Petra Gastmeier, Renate Krüger, Pia-Alice Hoppe, Sylke Schneider-Burrus, Irina Zuschneid, Nicoletta Wischnewski, Jennifer Bender, Franziska Layer, Michaela Niebank, Carmen Scheibenbogen, Leif G. Hanitsch

**Affiliations:** 1 Institute of Hygiene and Environmental Medicine, Charité Universitätsmedizin Berlin, Berlin, Germany; 2 PVL Workgroup, Charité Universitätsmedizin Berlin, Berlin, Germany; 3 Department of Pediatric Pulmonology and Immunology, Charité Universitätsmedizin Berlin, Berlin, Germany; 4 Department of Dermatology and Allergy, Charité Universitätsmedizin Berlin, Berlin, Germany; 5 Charlottenburg-Wilmersdorf Health Department, Berlin, Germany; 6 Robert Koch Institute, Wernigerode, Germany; 7 Department of Internal Medicine/Infectious Diseases and Pulmonary Medicine, Charité Universitätsmedizin, Berlin, Germany; 8 Institute of Medical Immunology, Charité Universitätsmedizin Berlin, Berlin, Germany; Universitatsklinikum Munster, GERMANY

## Abstract

**Objectives:**

We report on an outbreak of skin and soft tissue infections (SSTI) among kindergarten families. We analyzed the transmission route and aimed to control the outbreak.

**Methods:**

The transmission route was investigated by nasal screening for Panton-Valentine leukocidin (PVL)-producing *Staphylococcus aureus* (PVL-SA), subsequent microbiological investigation including whole genome sequencing and a questionnaire-based analysis of epidemiological information. The control measures included distribution of outbreak information to all individuals at risk and implementation of a *Staphylococcus aureus* decontamination protocol.

**Results:**

Individuals from 7 of 19 families were either colonized or showed signs of SSTI such as massive abscesses or eye lid infections. We found 10 PVL-SA isolates in 9 individuals. In the WGS-analysis all isolates were found identical with a maximum of 17 allele difference. The clones were methicillin-susceptible but cotrimoxazole resistant. In comparison to PVL-SAs from an international strain collection, the outbreak clone showed close genetical relatedness to PVL-SAs from a non-European country. The questionnaire results showed frequent travels of one family to this area. The results also demonstrated likely transmission via direct contact between families. After initiation of *Staphylococcus aureus* decontamination no further case was detected.

**Conclusions:**

Our outbreak investigation showed the introduction of a PVL-SA strain into a kindergarten likely as a result of international travel and further transmission by direct contact. The implementation of a *Staphylococcus aureus* decontamination protocol was able to control the outbreak.

## Introduction

*Staphylococcus aureus* is the most common cause of community-acquired skin and soft tissue infections (SSTI) [1]. In particular, the development of massive skin abscesses in otherwise healthy individuals is associated with certain strains of community-acquired *S*. *aureus* [2, 3]. These strains very often carry a gene-encoding Panton-Valentine leukocidin (PVL). PVL is a porin-like exotoxin that lyses leukocytes and is associated with dermonecrosis in the animal model [4]. In the USA, most isolates from outbreaks resulting from community-acquired Methicillin-resistant *S*. *aureus* (CA-MRSA) carry the PVL gene. In Europe, similar outbreaks are often associated with strains of Methicillin-susceptible *S*. *aureus* (CA-MSSA) [5]. However, both types of outbreaks occur within similar settings. They generally include close physical contact, the sharing of clothes such as sports or work clothes, or sauna visits [2, 3]. Here, we report an outbreak of pyoderma resulting from PVL-positive MSSA and its control among children in an urban kindergarten and their families in Berlin, Germany.

## Methods

This work evolved from a cooperation during an outbreak investigation. Only secondary data from the outbreak scenario was used to conduct this analysis. Written informed consent was obtained from all participants. The provided data was strictly anonymized for analysis and publication.

Exclusively RL and LH had access to the original data from the questionnaires and access to identifying information. As this work arose from routine infection control analyses, the Charité institutional review board waived the necessity of a previous ethics approval (Ethikausschuss 4 Campus Benjamin Franklin, process number EA4/112/17).

In March 2016, we were informed of a cluster of pyoderma cases among children who attended the same kindergarten. We established a multidisciplinary outbreak team to treat affected individuals and started a comprehensive investigation to control the outbreak. We arranged an information meeting with all families in order to discuss transmission routes and possible control measures. We performed a screening for nasal PVL-positive *S*. *aureus* (PVL-SA) colonization on all the kindergarten children and teachers. PVL-SA was detected by bacterial culture and subsequent PCR [6].

The genetical relatedness of the PVL-SA strains was investigated by whole genome sequencing (WGS) at the German National Reference Center (NRC) for Staphylococci and Enterococci at the Robert Koch Institute. Illumina short read sequencing and subsequent bioinformatics analyses using the SeqSphere+ software suite (Ridom, Münster, Germany) were utilized for this analysis.

DNA was isolated utilizing the DNeasy Blood 48 and Tissue Kit (Qiagen), followed by library preparation (Nextera XT Library Prep Kit, Illumina) and Illumina short read sequencing in paired-end on a MiSeq instrument (2x300 bp). Trimmed reads were assembled de novo using the a5-miseq algorithm (Coil et al., Bioinformatics, 2015) and contigs were analyzed by the SeqSphere+ software suite (Ridom, Münster, Germany). Strain relatedness based on core genome MLST (cgMLST) data was inferred from 1790 loci. Allelic differences are defined as novel alleles allocated due to the presence of SNPs, insertions or deletions.

We developed a questionnaire that was distributed among all families of children in the kindergarten (S1 Questionnaire). We inquired about the following subjects: skin infections among family members, chronic skin diseases or other underlying diseases, age, sex, contact with other kindergarten families outside of the kindergarten, pets, and travel abroad.

## Results

Initially, we knew of three symptomatic children who had had severe abscesses during the previous weeks. This included recurrent abscesses on fingers, necks, knees, thighs, or ears. In all cases, *S*. *aureus* expressing resistance to cotrimoxazole was isolated. The production of PVL in the *S*. *aureus* isolates was examined only in one case and found positive. We suspected an outbreak and notified the local health authorities. Twenty-three children from 19 families attended the kindergarten. We found two asymptomatic children carrying PVL-positive *S*. *aureus*. All isolates were methicillin-susceptible but cotrimoxazole-resistant. In addition, all household contacts of symptomatic children or children with PVL-SA carriage were contacted and screened for nasal or pharyngeal colonization.

**Fig 1:**
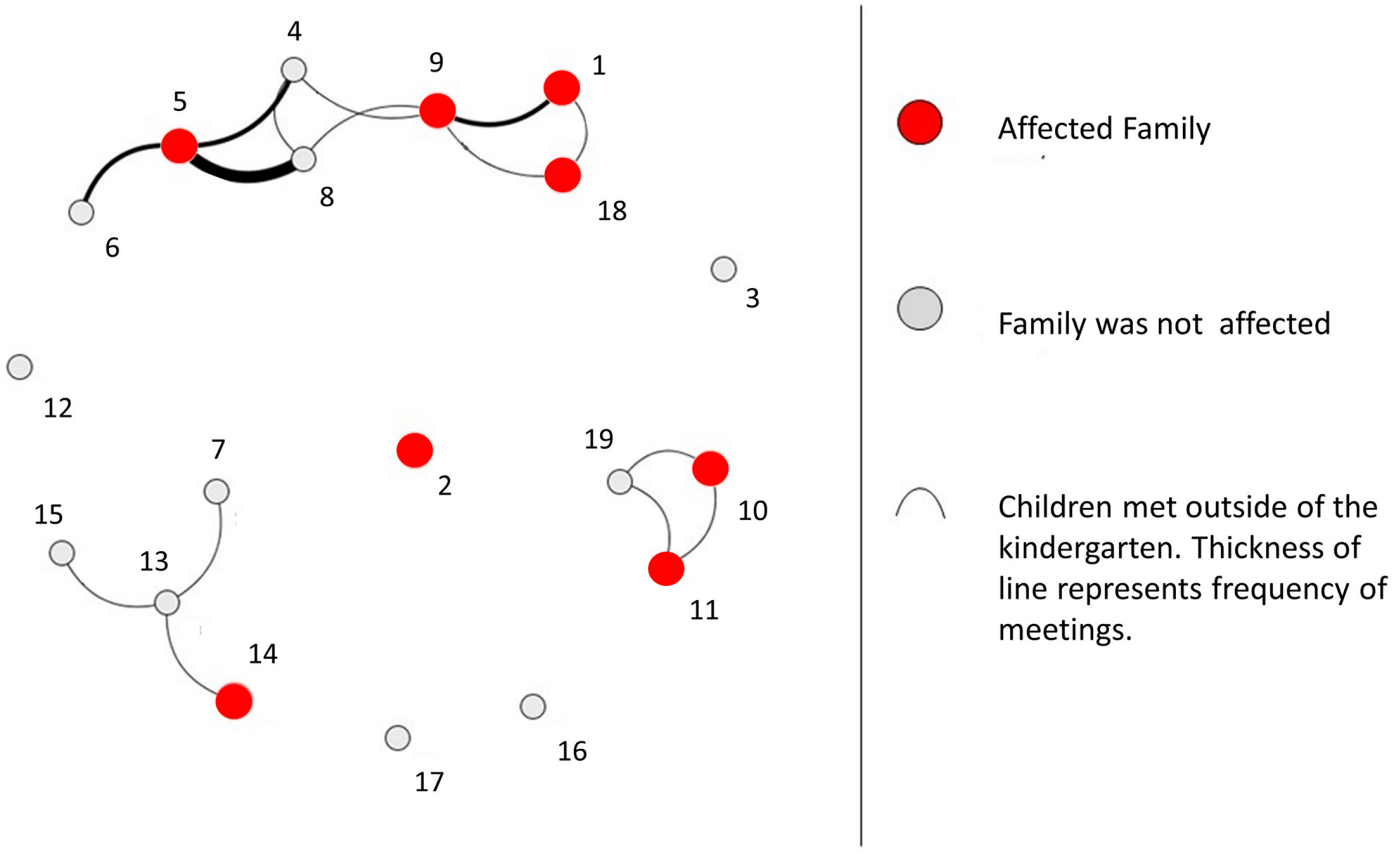
Visualization of the social network among kindergarten families outside of the kindergarten.

The response rate of the questionnaire was 79% (15 of 19 families). The answers showed that within the previous 12 months, members of two additional families had been diagnosed with eyelid abscesses. We defined families with either PVL-SA-colonized or symptomatic members as affected or case families. Based on the questionnaire’s information on play dates outside the kindergarten, we created a social network visualization (Fig 1) (The figure was created using open access software (http://cuttlefish.sourceforge.net/). The illustration shows the frequency of contacts between affected and unaffected families. It describes a cluster of play dates among case families and suggests that the predominant transmission route was most likely direct contact between the children.

We initiated a 5-day course of *S*. *aureus* decolonization for the affected families (symptomatic regardless colonization status or PVL-SA carrier) that included all of the family members who shared a single apartment. The core decolonization protocol consisted of nasal mupirocin or octenidine ointment three times per day; twice-daily mouthwash with octenidine or chlorhexidine; and, daily washing of the body with octenidine or chlorhexidine. Moreover, we recommended changing all personal care products including toothbrushes, combs and skin care products, and washing all clothes, towels and bed linens at 60°C. However, in the control screening-one week after the first decolonization-we found one positive child and one positive kindergarten teacher. We repeated the decolonization protocol for the remaining carriers a second time, followed by a control screening (2 weeks later) and a third repetition followed by a screening only for the remaining carriers (S1 Table). During a follow-up period of ten months, the affected families did not report any further skin or soft tissue infections. No additional cases occurred in the kindergarten.

**Fig 2:**
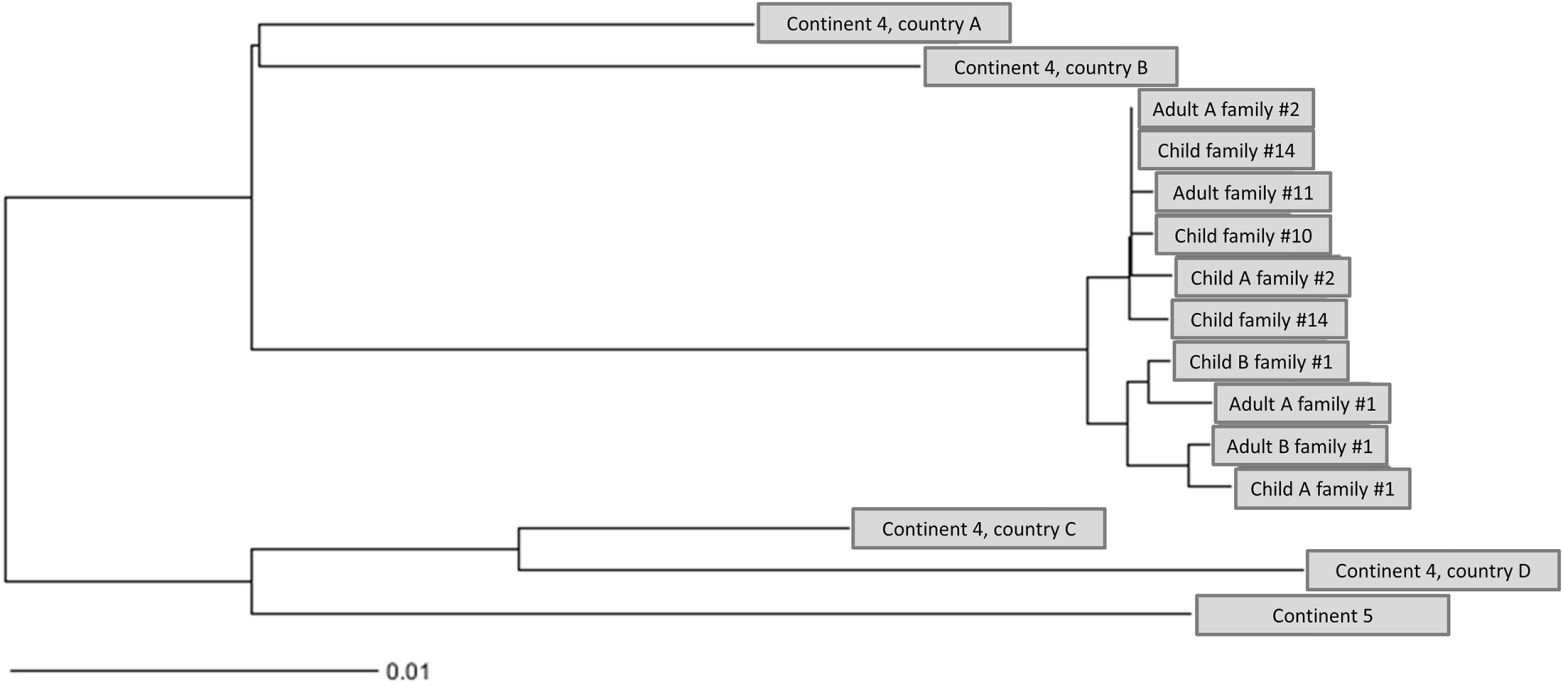
Allele-based neighbor-joining (NJ) tree, using cgMLST profiles of *S*. *aureus* from the suspected outbreak (10 isolates from 9 individuals) and from the NRCs strain collection (countries A-D).

Whole genome sequencing (WGS) was performed on 10 screening isolates of 9 individuals from 5 families. Sequence type (ST) 1633 and spa-type t355 was verified for all outbreak isolates. While MLST is based on 7 housekeeping genes only, further sub-differentiation with enhanced discriminatory power was achieved by extracting cgMLST types (CT) considering a total of 1790 loci. Thereby, CT 3142 was determined for the outbreak isolates which strengthens the hypothesis of strain transmission between various family members and unrelated individuals. As an example and as inferred from pairwise comparisons of the outbreak isolates, a maximum of 11 allele difference was detected between child A from fam#1 and adult A from fam#2 (Fig 2). In order to infer evolutionary relatedness, we completed the sample set with isolates from the strain collection of the German NRC for Staphylococci. They were acquired from pyoderma cases from 5 different countries on 2 continents, exhibiting the following characteristics: MSSA, PVL-positive, spa-type t355. The outbreak cluster was clearly separated from the closest relative by 68 alleles (Fig 2). This strain (ST152) originated from country A, outside of Europe. Interestingly, and concordant to the answers given in the above mentioned questionnaire survey, members of family #1 travel several times per year to this country. The hypothesis was further examined by comparing PVL-encoding genes and flanking genetic regions hence verifying a close relationship between country A and the outbreak isolates (not shown). Unfortunately, due to data protection of the respective family we are obliged to use a pseudonym for the respective country.

## Discussion

This outbreak of PVL-SA-associated skin infection was most likely following introduction of the pathogen via international travel and subsequent transmission via direct contact. WGS of the screening isolates showed a genetically close relationship between the outbreak strain and an often-visited country of one family (family #1). International travel has been described as risk factor of PVL-SA acquisition and introduction into communities [7, 8].

Whereas skin infections on trunk and extremities are the most common sites, some patients showed signs of periorbital skin infection. This is rare, but has been previously described as PVL-SA-associated infections [2]. Direct contact is the most likely route of PVL-SA transmission [2]. As our data showed a clustering pattern of PVL-SA cases among children that often met for play dates, our data underlines this assumption.

Our results also show that some colonized individuals can develop serious cases of pyoderma while others merely remain colonized without signs of skin infection (e.g. family #1). This goes along with the meta-analysis by Shallgross et al. [2]. Moreover, our survey did not reveal any association with preexisting skin conditions in this family. This indicates a distinct pathogen-host interaction that allows for infection in some individuals. However, a study by Hermos et al. showed that simply high levels of antibodies to PVL components LukF and LukS are not associated with resistance to SSTI [9]. Future studies on PVL-SA colonized individuals without history of skin infection are necessary to provide explanations for this phenomenon.

*S*. *aureus* decolonization treatments interrupted further transmission and enabled control of the outbreak. This is reported to be highly effective in clinical patients while having a reduced effect in outpatients [2, 3].

## Conclusion

We investigated an outbreak of skin infections among families of kindergarten children. WGS analysis together with the results of a questionnaire showed the introduction of a PVL-SA strain into a kindergarten. This occurred likely as a result of international travel and further transmission by direct contact. The implementation of a *S*. *aureus* decolonization protocol for affected families led to the control of the outbreak.

## Supporting information

S1 Table Results of the nasal screening for PVL-positive *S*. *aureus*. *, abscess within the last 12 months.
(DOC)

S1 Questionnaire (DOC)
