# Supplementary material for: Pyoderma outbreak among kindergarten families: Association with a Panton-Valentine leukocidin (PVL)-producing S. aureus strain
Source: PLoS One. 2017 Dec 19;12(12):e0189961. doi: 10.1371/journal.pone.0189961 (PMC5736205; doi:10.1371/journal.pone.0189961)
Supplement: S1 Table — *, abscess within the last 12 months. (DOC) [file pone.0189961.s001.doc]

S1 Table: Results of the nasal screening for PVL-positive *S. aureus*. *, abscess within the last 12 months.

|  |  | **Nasal Screening for PVL-positive *S. aureus*** | | |
| --- | --- | --- | --- | --- |
|  |  | **1^st^** | **2^nd^** | **3^rd^** |
|  | **Children included** | 19 | 20 | 18 |
|  | **Teachers included** | 0 | 9 | 6 |
| **PVL detection** | **Child from family No.10*** | - | - | - |
|  | **Child from family No.2*** | - | - | - |
|  | **Child from family No.18*** | - | - | - |
|  | **Child from family No.14†** | + | + | + |
|  | **Child from family No.1** | + | - | + |
|  | **Teacher A** | not screened | + | - |

All family members of affected families underwent topical *S. aureus* decolonization after each screening. *, patient with SSTI. †, child was eventually decolonized after undergoing a third round of decolonization in combination with antibiotic therapy.
