# Supplementary material for: Pyoderma outbreak among kindergarten families: Association with a Panton-Valentine leukocidin (PVL)-producing S. aureus strain
Source: PLoS One. 2017 Dec 19;12(12):e0189961. doi: 10.1371/journal.pone.0189961 (PMC5736205; doi:10.1371/journal.pone.0189961)
Supplement: S1 Questionnaire — (DOC) [file pone.0189961.s002.doc]

| **Members of the family** | | | | | | |
| --- | --- | --- | --- | --- | --- | --- |
| **Father/mother/ child no.** | **Given**  **name** | **Family name** | **Birth date** | **Sex (M/W)** | **Does the affected attend kindergarten** | **Was a PVL-SA screening performed?** |
| *Example* | *Leon* | *Müller* | *29.08.2013* | *M* | *no* | *no* |

| **No.** | **Question** |
| --- | --- |
| **01.1** | Has your child had a skin infection, e.g. an abscess, during the last 12 months? |
| **01.2** | Was the abscess surgically drained? |
| **01.3** | Was a hospital stay necessary because of this? |
| **01.4** | Did the child receive antibiotics? |
| **02** | Have there been other cases of skin infections in your family during the last 12 months? |
| **03** | Does your child have a chronic skin condition, e.g. atopic dermatitis or psoriasis? |
| **04** | Does your child have any other chronic conditions, e.g. diabetes, bronchial asthma, hay fever? |
| **05** | Do any other persons who are not part of your household, e.g. grandparents, have frequent close contact with your child ? |
| **06** | How many people live in your household? |
| **07** | How big is your apartment in square meters? |
| **08** | Have other family members had abscesses during the last 12 months? |
| **09** | Have any family members had contact with other persons with skin abscesses? |
| **09** | Are there pets in your household? |
| **10.1** | Has your child played with children from the affected kindergarten outside the kindergarten during the last 6months (play dates)? |
| **10.2** | If so, who was this child and how many times did the children play together during the last 6 months? |
| **11** | Has your child been abroad during the last 12 months? |
| **12** | Have any of your family members been abroad during the last 12 months? |
| **13** | What is/are the main language(s) spoken among your family members? |
